# Development and Optimization of the Novel Fabrication Method of Highly Macroporous Chitosan/Agarose/Nanohydroxyapatite Bone Scaffold for Potential Regenerative Medicine Applications

**DOI:** 10.3390/biom9090434

**Published:** 2019-09-01

**Authors:** Paulina Kazimierczak, Krzysztof Palka, Agata Przekora

**Affiliations:** 1 Department of Biochemistry and Biotechnology, Medical University of Lublin, Chodzki 1, 20-093 Lublin, Poland; 2 Department of Materials Engineering, Lublin University of Technology, Nadbystrzycka 36, 20-618 Lublin, Poland

**Keywords:** porosity, biocompatibility, cytotoxicity, micro-CT analysis, compressive strength, Young’s modulus

## Abstract

Bone scaffolds mimicking the three-dimensional bone structure are of essential importance for bone regeneration. The aim of this study was to develop and optimize the production method of highly macroporous bone scaffold composed of polysaccharide matrix (chitosan–agarose) reinforced with nanohydroxyapatite. The highly macroporous structure was obtained by the simultaneous application of a gas-foaming agent and freeze-drying technique. Fabricated variants of biomaterials (produced using different gas-foaming agent and solvent concentrations) were subjected to porosity evaluation and compression test in order to select the scaffold with the best properties. Then, bioactivity, cytotoxicity, and cell growth on the surface of the selected biomaterial were assessed. The obtained results showed that the simultaneous application of gas-foaming and freeze-drying methods allows for the production of biomaterials characterized by high total and open porosity. It was proved that the best porosity is obtained when solvent (CH_3_COOH) and foaming agent (NaHCO_3_) are applied at ratio 1:1. Nevertheless, the high porosity of novel biomaterial decreases its mechanical strength as determined by compression test. Importantly, novel scaffold is non-toxic to osteoblasts and favors cell attachment and growth on its surface. All mentioned properties make the novel biomaterial a promising candidate to be used in regenerative medicine in non-load bearing implantation sites.

## 1. Introduction

Over recent decades, there has been a growing interest in bone tissue engineering as an alternative strategy to regenerative medicine. Scaffolds for bone regeneration should possess appropriate structural features (e.g., surface topography, mechanical properties, high porosity) and biological requirements (e.g., non-toxicity, biocompatibility, bioactivity, biodegradability). Moreover, biomaterials used in bone tissue engineering should exhibit a microstructure mimicking highly porous and three-dimensional (3D) bone structure, which is essential for acceleration of the regeneration process and implant vascularization [1,2,3]. 

Typical tissue engineered products (TEPs) are composed of a 3D porous scaffold which is often loaded with stem cells and/or growth factors [3,4,5]. The porous structure of the scaffold is a critical feature because it ensures space for migration and proliferation of the cells, vascular ingrowth, as well as good oxygenation, nutrient diffusion and waste products elimination [3,6,7]. Moreover, appropriate pore size plays an important role in the bone regeneration process. According to the available literature, the minimum diameter of pores allowing for good implant osseointegration should be approximately 100 µm [6]. In the field of engineering of biomaterials, porosity of the scaffold is defined as microporosity when pore diameter is less than 2 µm, as mesoporosity when pore diameter is in the range of 2–50 µm, and as macroporosity when pore diameter is higher than 50 µm [6,8,9]. Considering the connection of the solid material structure with the space outside (air), the porosity of the biomaterials may be categorized as closed (without the connection into the space outside the material and without access to fluids) and open (with the connection into the space outside the material and having access to fluids). Total porosity of biomaterials is a sum of closed and open porosity [3,9,10]. Importantly, TEPs for regenerative medicine applications should primarily possess highly interconnected and open porosity, which are considered as the most significant types of porosity, enabling fluids to flow through the biomaterial and supporting bone tissue ingrowth and blood vessel formation [3,7,9,11]. 

In the field of engineering of biomaterials, it is very important to select a suitable method for the introduction of pores into the structure of the scaffold in order to obtain highly porous biomaterial with relatively good mechanical properties. Among the various techniques that exist for the fabrication of porous biomaterials, the methods of porogen-leaching, freeze-drying and gas-foaming—which are relatively simple and cost-effective—appear to be the most commonly used [8,12,13,14,15,16,17,18]. However, according to the available literature, researchers apply these mentioned techniques separately, often obtaining high porosity with a majority of closed pores and inadequate pore interconnectivity [6,12,14]. For example, Li et al. [17] used the gas-foaming agent method for the production of porous material made of HA and polymethylmethacrylate (PMMA) resin. Similarly, Kar et al. [16] developed porous chitosan-modified montmorillonite–hydroxyapatite composite scaffolds using sodium bicarbonate as a gas-foaming agent. Kim et al. [18] produced a nanohydroxyapatite–alginate–chitosan composite via freeze-drying technique. Likewise, Felfel et al. [14] fabricated 3D chitosan–agarose scaffolds by application of the lyophilization process. Other popular methods for the fabrication of porous scaffolds include complex and advanced approaches such as 3D printing [19,20,21,22,23] and electrospinning [24,25,26]. 

The aim of this study was to develop and optimize the simple production method of novel macroporous bone scaffold composed of polysaccharide matrix (chitosan–agarose) reinforced with bioceramics (hydroxyapatite nanopowder). To obtain a highly porous structure characterized by primarily open and interconnected porosity, a gas-foaming agent was combined with the freeze-drying method. To conduct a foaming process (carbon dioxide release), the sodium bicarbonate (NaHCO_3_) was subjected to the reaction with the acetic acid (CH_3_COOH): NaHCO_3_ + CH_3_COOH → CH_3_COONa + H_2_O + CO_2_↑ and also to a high temperature (95 °C): 2 NaHCO_3_ → Na_2_CO_3_ + H_2_O + CO_2_↑. In order to obtain a desirable macroporosity, optimization of the CH_3_COOH and NaHCO_3_ concentrations was performed during the fabrication process. It is worth noting that the composition of developed biomaterial and the method for its production are described in Polish patent application no. P.426788. Produced scaffolds were subjected to porosity evaluation and compression test in order to select the scaffold with the best properties. Then, bioactivity, cytotoxicity, and cell growth on the surface of the selected biomaterial were assessed.

## 2. Materials and Methods

### 2.1. Optimization of the Scaffold Fabrication Method

**Figure 1:**
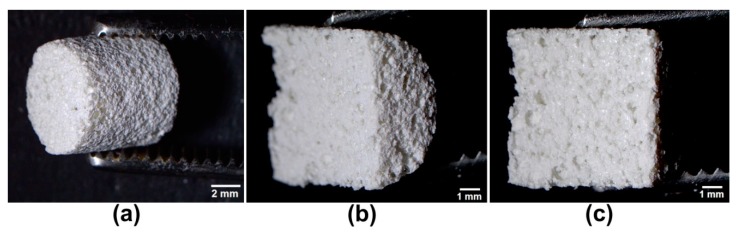
Stereoscopic microscope images presenting microstructure of the mat_2_2 scaffold: (**a**) produced scaffold (scale bar = 2 mm); (**b**,**c**) longitudinal sections representing the exact middle of the mat_2_2 (scale bar = 1 mm).

To optimize the production process of the macroporous scaffold, different variants of biomaterials were produced using various concentrations of solvent (CH_3_COOH) and gas-foaming agent (NaHCO_3_). Biomaterials were synthesized by suspending of 2% *w/v* chitosan (75–85% deacetylation degree, viscosity ≤300 cP, 50–190 kDa molecular weight, Sigma-Aldrich Chemicals, Warsaw, Poland) and 5% *w/v* agarose (low EEO, gel point 36 ± 1.5 °C, Sigma-Aldrich Chemicals, Warsaw, Poland) in CH_3_COOH (Avantor Performance Materials, Gliwice, Poland) applied in the concentration range of 0.5–2% *v/v*. Then, the obtained blend was mixed with 30% *w/v* nanohydroxyapatite (nanoHA, Sigma-Aldrich Chemicals, Warsaw, Poland) and NaHCO_3_ (Sigma-Aldrich Chemicals, Warsaw, Poland), applied in the concentration range of 0.5–2% *w/v*, as shown in Table 1. The resultant paste was transferred into cylinder-shaped forms, which were subsequently heated for 15 min in a water bath at 95 °C. Then, the samples were cooled, frozen and lyophilized (LYO GT2-Basic, SRK Systemtechnik GmbH, Riedstadt, Germany). The biomaterials were neutralized in 1% *w/v* sodium hydroxide solution (Avantor Performance Materials), washed with deionized water, and dried at room temperature for 24 h. Control sample was produced without the use of gas-foaming agent and the control biomaterial was air dried instead of freeze-drying. The microstructure of developed porous scaffold (mat_2_2) was visualized using a stereoscopic microscope (Olympus SZ61TR, Olympus Polska Sp. z o. o., Warsaw, Poland) (Figure 1). Prior to the cell culture experiments, the samples were sterilized using ethylene oxide.

### 2.2. Porosity Determination

The porosity of the biomaterials was studied by micro-computed tomography—micro-CT (Skyscan 1174, Bruker microCT, Kontich, Belgium) with 12 µm voxel resolution. Reconstruction of the 360 cross-section images representing the middle of the samples was conducted using the NRecon 1.7.1.6 software (Bruker microCT, Kontich, Belgium), whereas porosity (closed, open, total) and pore diameter assessment was performed using the CTAnalyser 1.17.7.2 software (Bruker microCT, Kontich, Belgium) which determines porosity in the whole selected 3D volume of interest (VOI).

### 2.3. Compression Test

The compressive strength of the cylinder-shaped samples (8 mm in diameter and 8 mm in length) was measured using an Autograph AG-X plus (Shimadzu Europa GmbH, Duisburg, Germany) testing machine at crosshead moving speed 10 mm/min with the load cell accuracy of 0.1 N. The compressive stress was calculated at 30% of strain. The obtained data also allowed for the evaluation of Young’s modulus values.

### 2.4. Bioactivity Test

A bioactivity test was performed for the scaffold to reveal the best structural properties. Evaluation of apatite-forming ability on the surface of the biomaterial was conducted according to the ISO 23317:2007 procedure. Disc-shaped biomaterial samples (3 mm thick and 8 mm in diameter) were immersed in a simulated body fluid (SBF, pH 7.4), which is characterized by ion concentrations similar to human blood plasma simulating in vivo conditions. The samples were incubated in SBF for 14 and 28 days at 37 °C, then the specimens were rinsed with deionized water, and dried in a desiccator without heating. Dried samples were sputtered under a high vacuum with gold layer (20 nm) and subjected to examination of their surfaces with a scanning electron microscope (SEM, Zeiss ULTRA plus) equipped with Octane Pro EDS detector (EDAX) (Carl Zeiss Microscopy, LLC, White Plains, New York, USA). Obtained EDS data allowed for calculation of the Ca/P atomic ratio to confirm the presence of apatite precipitates on the surfaces of the samples. Additionally, during incubation of the biomaterial in SBF, samples of the liquid were collected to estimate the changes in concentrations of Ca^2+^ and HPO_4_^2−^ ions. The ion concentrations were evaluated by colorimetric methods using appropriate commercial detection kits (BioMaxima, Lublin, Poland).

### 2.5. Cell Culture Experiments

Cell culture experiments were performed for the scaffold revealing the best structural properties. Mouse calvarial preosteoblast cell line (MC3T3-E1 Subclone 4, ATCC-LGC, standards, Teddington, UK) was used to assess cytotoxicity of the fabricated bone scaffold and cell growth on its surface. MC3T3-E1 cells were cultured in alpha MEM medium (Gibco, Life technologies, Carlsbad, California, USA), supplemented with 10% fetal bovine serum (Pan-Biotech GmbH, Aidenbach, Bavaria, Germany), 100 μg/mL streptomycin (Sigma-Aldrich Chemicals, Warsaw, Poland), 100 U/mL penicillin (Sigma-Aldrich Chemicals, Warsaw, Poland), and maintained at 37 °C in 5% CO_2_ in air atmosphere.

#### 2.5.1. Cytotoxicity Tests

The cytotoxicity assessment was performed according to ISO 10993-5:2009 by indirect method using 24-h extracts of the biomaterial prepared as described previously [12]. MC3T3-E1 cells were seeded into 96-multiwell plates at a concentration of 2 × 10^4^ cells per well and cultured for 24 h. Then, the culture medium was replaced with the extract of the scaffold and polypropylene (PP) material, which served as a negative control of cytotoxicity. After 24 h, 48 h, and 72 h of cell incubation at 37 °C, MTT (Sigma-Aldrich Chemical, Warsaw, Poland) colorimetric test was performed to evaluate cell viability. The MTT results were presented as the percentage of absorbance value obtained with the PP material (negative control of cytotoxicity considered as 100% viability).

The cytotoxicity of the scaffold was also evaluated by direct contact test via staining of the osteoblasts cultured on the surface of the investigated biomaterial. Before cell seeding, scaffold discs (2 mm thick and 8 mm in diameter) were placed in a 48-multiwell plate and preincubated overnight in a complete culture medium. Then, 1 × 10^5^ cells were seeded directly onto the scaffold discs in 500 µl of culture medium and the osteoblasts were cultured for further 48 h at 37 °C. The MC3T3-E1 cells were stained using a Live/Dead Double Staining Kit (Sigma-Aldrich Chemical, Warsaw, Poland) and analyzed using a confocal laser scanning microscope (CLSM, Olympus Fluoview equipped with FV1000, Olympus Polska Sp. z o. o., Warsaw, Poland).

#### 2.5.2. Cell Growth on the Surface of the Scaffold

In order to determine the growth of osteoblasts on the surface of the biomaterial, 2.5 × 10^4^ cells were seeded directly onto the discs in 500 µL of culture medium. The osteoblasts were cultured for 2 and 4 days at 37 °C and then the cells were fixed with 3.7% paraformaldehyde (Sigma-Aldrich Chemicals, Warsaw, Poland), permeabilized with 0.2% Triton X-100 (Sigma-Aldrich Chemicals, Warsaw, Poland), and blocked with 1% bovine serum albumin (Sigma-Aldrich Chemicals, Warsaw, Poland). The cytoskeletal filaments (F-actin) and nuclei were stained for 30 min at room temperature with AlexaFluor635-conjugated phallotoxin (Invitrogen, Carlsbad, California, CA, USA) and DAPI (Sigma-Aldrich Chemicals, Warsaw, Poland), respectively. Stained cells were visualized by CLSM.

### 2.6. Statistical Analysis

The data were presented as mean values ± standard deviation (SD) and the tests were repeated in at least three independent experiments (*n* = 3). Statistical analysis of the results was carried out using an unpaired *t*-test or One-way ANOVA followed by Tukey’s test with a significance considered at *p* < 0.05 (GraphPad Prism 8.0.0 software, GraphPad Software Inc., California, CA, USA).

## 3. Results and Discussion

### 3.1. Porosity Determination

**Figure 2:**
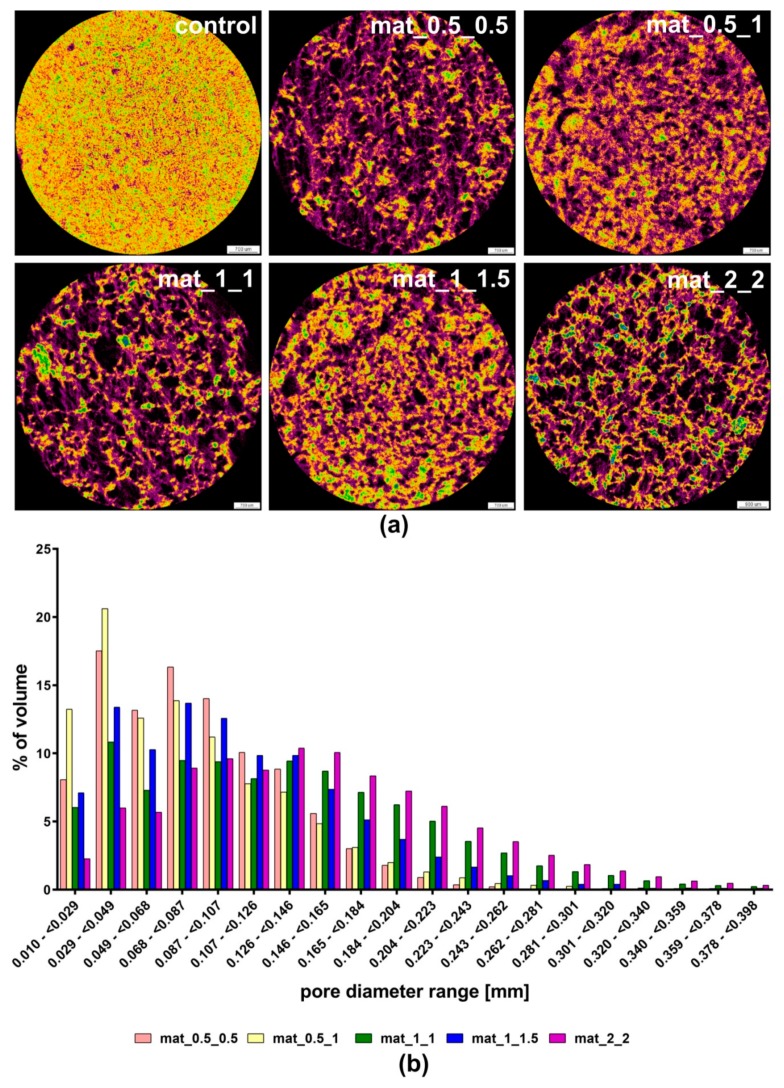
Pores size and their distribution within the structure of biomaterials determined with the use of micro-CT: (**a**) cross-section images of the scaffolds (black color—pores; violet color—polysaccharide matrix, yellow/orange/green/blue colors—nanoHA); (**b**) chart presenting pore share of different diameters in the structure of the scaffolds.

Evaluation of porosity and mechanical properties of fabricated scaffolds was critical for the optimization of the production process. The microstructure of different variants of the produced biomaterials was characterized using micro-CT. Comparison of the percentage of each type (open, closed, total) of porosity between the scaffolds and the control sample demonstrated that the novel production method applied here (combining gas-foaming and freeze-drying) allowed for highly porous structures of the biomaterials to be obtained (Table 2, Figure 2). The results showed that materials produced using CH_3_COOH and NaHCO_3_ at a ratio of 1:1 (mat_0.5_0.5, mat_1_1, and mat_2_2) exhibited higher total and open porosity compared to the samples fabricated using a solvent and gas-foaming agent at a ratio of 1:2 (mat_0.5_1) and 1:1.5 (mat_1_1.5), indicating that the excess of the gas-foaming agent in the reaction mixture negatively affected CO_2_ release and pore formation. Moreover, it was observed that the higher solvent and gas-foaming agent concentrations were applied, the higher porosity was obtained. Therefore, mat_2_2 was characterized by the highest total (approximately 70%) and open (approximately 50%) porosity among all other scaffolds. It should be noted that open and interconnected porosities of the biomaterial are crucial for bone tissue formation, as they facilitate migration and proliferation of osteoblasts/stem cells as well as allow for new blood vessel formation within the implant [3,4,5,7]. It is also worth emphasizing that porosity of the mat_2_2 had comparable value to the porosity of trabecular bone, which was estimated to be in the range of 50–90% [4]. According to the literature, a porosity degree of the bioceramics-based scaffolds occurring in the range of 40–80% is desirable, as it shows a positive effect on bone formation process, whereas a porosity higher than 80% may cause the untimely collapse of the biomaterial at the implantation site [13].

The cross-section images of fabricated scaffolds obtained with micro-CT scanning showed their highly macroporous and interconnected structure (Figure 2a). Importantly, the mat_2_2 revealed a more uniform interconnected pore network and pore distribution compared to other scaffolds. The images clearly showed that the materials produced using the solvent and gas-foaming agent at ratio 1:1 exhibited higher porosity than the samples fabricated using an excessive amount of NaHCO_3_. Micro-CT analysis also demonstrated that biomaterials produced using 0.5% *v/v* CH_3_COOH had a high share of small pores (10–70 μm), whereas scaffolds prepared using a greater concentration of solvent (1% or 2% *v/v*) revealed a high share of large pores (>150 μm) (Figure 2b). Furthermore, samples fabricated using the solvent and gas-foaming agent at ratio 1:1 (mat_1_1 and mat_2_2) exhibited the highest share of large pores (150–400 μm) among all investigated biomaterials.

Importantly, it has been shown that biomaterials possessing large pores, with a diameter in the range of 100–500 µm, can provide optimal osseointegration with the host tissue since they facilitate scaffold cellular infiltration resulting in better bone ingrowth [15,27]. Materials that have pores with a diameter lower than 75 µm support fibrous tissue formation, which is an undesirable phenomenon [6]. Nevertheless, microporosity (a pore diameter <2 µm) also has biomedical importance because it makes the implant surface area larger and introduces greater surface roughness, supporting protein adsorption and thereby osteoblast attachment and proliferation [28]. Although pore size is an important factor for good implant osseointegration, if the scaffold does not reveal interconnectivity of pores, then it is useless in tissue engineering applications. Thus, it is pore interconnectivity that is the most critical feature of the porosity, determining accelerated bone regeneration. However, the size of voids (interconnections) between the pores should be at least 100 µm in order to enable cell migration. Thus, the ideal scaffold should have 50–90% macroporosity (>100 µm) and 100% interconnectivity. It should not have pores with diameter lower than 75 µm and mesopores (2–50 µm) which are known to induce material encapsulation and its failure [6]. Microporosity (<2 µm) is not as important as macroporosity, but it is still desired since it has an impact on implant topography and its bioactivity [28].

### 3.2. Compression Test

The compression test demonstrated that the introduction of pores into the biomaterials structure significantly worsened the mechanical properties of the resultant samples, as compared to the control material (Table 3). All fabricated scaffolds revealed comparable compressive strength and Young’s modulus values which were in the range of 0.77–2.35 MPa and 6.04–11.79 MPa, respectively. The control sample showed compressive strength equal to 36.19 MPa and Young’s modulus value equal to 179.14 MPa. Importantly, although fabricated scaffolds revealed different degrees of porosity, no statistically significant differences in mechanical properties between the samples were observed. It should be noted that mechanical parameters of the human bone are much higher than values estimated for the presently investigated scaffolds, e.g., compressive strength value for trabecular bone is in the range of 1.9–12 MPa, whereas its Young’s modulus value is in the range of 0.1–0.5 GPa [29]. Low Young’s modulus values determined for produced scaffolds indicated their high elasticity, which is not a desired phenomenon when biomaterial is exposed to the mechanical load after its implantation. An elastic scaffold implanted in a load-bearing area may exert high physical pressure to the host bone and act as a mechanical stimulus to the osteoblasts, inducing excessive bone formation process and implant failure [10,30]. Therefore, based on the obtained results, it was concluded that the developed biomaterials may be applied in non-load bearing implantation sites or should be stabilized with screws, wires, pins or plates, in order to prevent failure of the scaffold after its implantation in the area exposed to mechanical load [31].

It should be noted that ceramic/polymer composite scaffolds are known to have low values of compressive strength and Young’s modulus, e.g., Li et al. [32] produced a PLLA–chitosan–hydroxyapatite scaffold showing compressive strength and Young’s modulus equal to 0.42 MPa and 1.46 MPa, respectively, whereas El-Meliegy et al. [33] developed a chitosan–dextran–nanoHA scaffold with a compressive strength of 0.6 MPa and Young’s modulus of 0.04 MPa. Micro-CT analysis and mechanical tests demonstrated that mat_2_2 had the highest degree of porosity (70%) and mechanical properties comparable to other porous scaffolds. Thus, the mat_2_2 sample was considered as the best candidate among all of the investigated scaffolds for bone tissue engineering applications. To prove its biomedical potential, mat_2_2 was subjected to a bioactivity test and basic cell culture experiments.

### 3.3. Bioactivity Test

**Figure 3:**
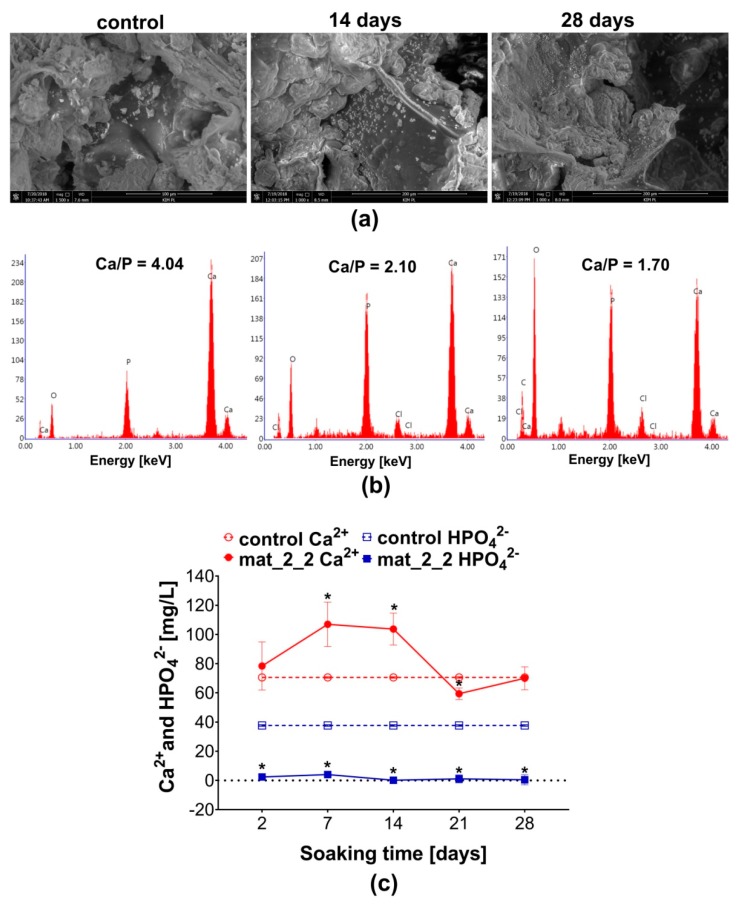
Bioactivity test: (**a**) SEM visualization of the surface of the mat_2_2 before its immersion in simulated body fluid (SBF) (control sample; magnification 1500×, scale bar = 100 µm) and after incubation in the SBF for 14 and 28 days (magnification 1000×, scale bar = 200 µm); (**b**) EDS spectra of apatite precipitates on the surface of the mat_2_2 along with calculated Ca/P atomic ratio; (**c**) Ca^2+^ and HPO_4_^2−^ concentrations in the SBF samples collected during incubation with the mat_2_2 (control Ca^2+^ and control HPO_4_^2−^ ion concentration in the fresh SBF; * statistically significant results compared to the control SBF, *p* < 0.05, unpaired *t*-test).

Formation of apatite crystals on the surface of the bone scaffold gives evidence of its high bioactivity, which is crucial for rapid bone regeneration and good implant osseointegration. After implantation of the biomaterial within the bone defect, its surface is covered with a thin layer of apatite (calcium phosphates) precipitates, which have a chemical composition and structure very similar to that of natural bone mineral. To predict the bioactivity of the novel bone scaffolds under in vitro conditions, biomaterials were incubated in SBF solution showing pH and ion concentrations similar to those in human blood plasma [34,35]. In this study, mat_2_2 was soaked in SBF solution for 14 and 28 days to evaluate its bioactivity (biomineralization in vitro). After 14-day incubation, SEM visualization showed precipitates with globular morphology on the surface of the scaffold (Figure 3a). The Ca/P atomic ratio calculated for observed precipitates was equal to 2.10 ± 0.19, suggesting that it was Ca-rich amorphous calcium phosphate (ACP) (Figure 3b). It is known that the short incubation of HA-based biomaterials in SBF induces interactions of negatively charged HA with Ca^2+^ ions occurring in the SBF, that results in the formation of Ca-rich ACP. It was also observed that longer incubation of biomaterials in SBF allows for the incorporation of more phosphate ions, reducing the Ca/P atomic ratio to the value comparable to natural HA and changing the ACP into bone-like apatite crystals [36]. Prolonged (28 days) incubation of mat_2_2 in the SBF not only resulted in the formation of greater amount of apatite crystals on its surface (Figure 3a), but also in the reduction of the Ca/P atomic ratio to the value of 1.70 ± 0.14 (Figure 3b), which is very close to the stoichiometric Ca/P value for hydroxyapatite (1.67) [37].

Bioactivity of mat_2_2 was confirmed by analysis of the Ca^2+^ and HPO_4_^2−^ concentrations in the SBF solution after incubation with the biomaterial (Figure 3c). On the 7th and 14th day of the experiment, a significant increase in Ca^2+^ concentration compared to the control SBF was observed. Then, on the 21st day significant decrease in calcium level was detected. Interestingly, complete uptake of HPO_4_^2−^ ions form the SBF was recorded throughout the whole experiment. Increased Ca^2+^ concentration in the SBF was most likely the result of the dissolution of nanoHA and release of high amount of Ca^2+^ ions, what caused supersaturation of the solution, followed by induction of apatite formation observed as there was a decline in Ca^2+^ level [37,38,39]. These kinds of fluctuations in ion concentrations are typical of apatite formation. Importantly, the high bioactivity of the mat_2_2 demonstrated here indicates its promising potential in biomedical applications.

### 3.4. Cell Culture Tests

**Figure 4:**
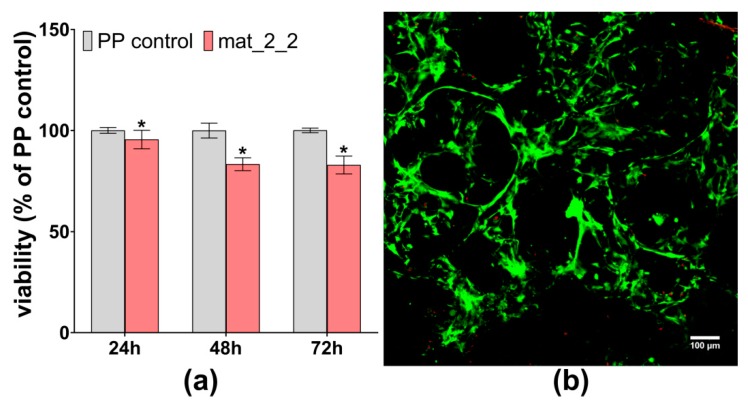
Cytotoxicity evaluation of the mat_2_2 scaffold against MC3T3-E1 cells: (**a**) MTT test after 24-h, 48-h, and 72-h exposure to the extracts (PP control—polypropylene extract as negative control of cytotoxicity; * statistically significant results compared to PP control, *p* < 0.05, unpaired *t*-test); (**b**) confocal laser scanning microscope (CLSM) image showing live/dead staining of cells cultured on the surface of the mat_2_2 scaffold (green fluorescence—viable cells, red fluorescence—dead cells, magnification 100×, scale bar = 100 µm).

To the evaluate biological response to a novel scaffold, the mat_2_2 was subjected to a cytotoxicity test and evaluation of osteoblast growth on its surface. The MTT test revealed non-toxicity of the mat_2_2 against MC3T3-E1 osteoblasts (Figure 4a). Viability of cells exposed for 24 h to the extract of the scaffold was near 100%. After 48-h and 72-h of exposure, cell viability was slightly reduced to approximately 83%. However, in accordance with ISO 10993-5, reduction in cell viability (by 100% extract of the sample) to the value lower than 70% indicates the toxicity of the biomaterial. It should be noted that it is commonly known that bioceramics-based biomaterials reveal ion reactivity causing changes in the ionic composition of the culture medium, what may consequently result in the reduction in cell viability [40,41,42]. Performed here, the bioactivity test showed that the produced novel scaffold exhibited high ion reactivity as it significantly changed the concentrations of Ca^2+^ and HPO_4_^2^^−^ ions in the SBF (Figure 3c). Therefore, the observed slight reduction in cell viability most likely was a result of changes in the ionic composition of the culture medium. As shown in Figure 4b, live/dead staining of osteoblasts cultured on the surface of the mat_2_2 confirmed the non-toxicity of the scaffold. The obtained CLSM image showed good adhesion of numerous viable (green fluorescence) osteoblasts to the surface of the scaffold and only few dead cells (red fluorescence).

**Figure 5:**
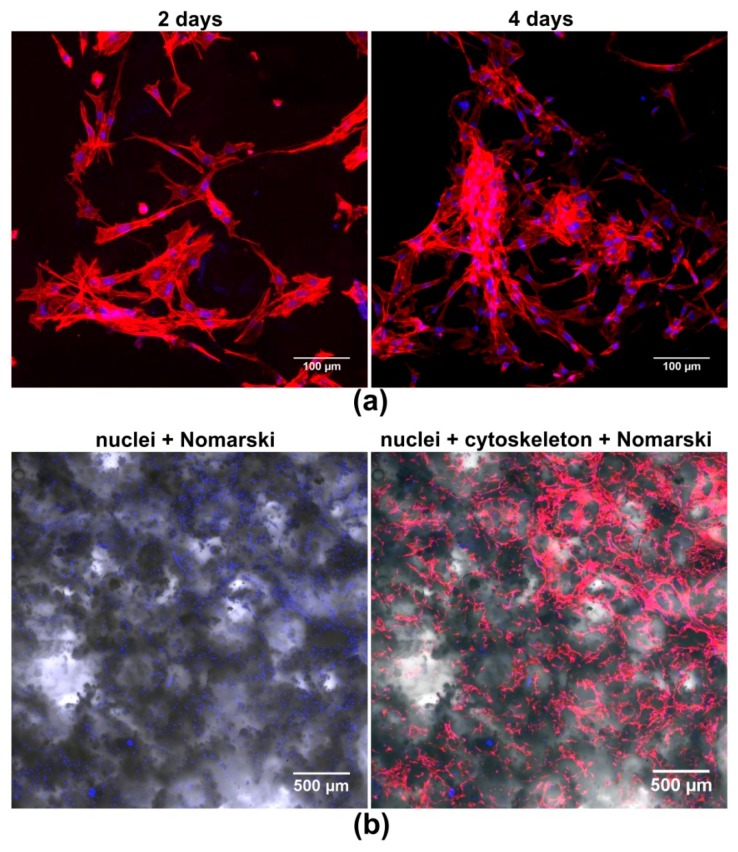
CLSM images presenting the growth of MC3T3-E1 osteoblasts on the surface of the mat_2_2 scaffold: (**a**) cell growth and morphology on the 2nd and 4th day, magnification 200×, scale bar = 100 µm; (**b**) lower magnification images showing structure of the biomaterial (thanks to applied Nomarski contrast) covered with osteoblasts, magnification 40×, scale bar = 500 µm (red fluorescence—cytoskeleton filaments, blue fluorescence—nuclei).

Strong cell adhesion to the scaffold surface is essential for good biocompatibility of the implant since it enables better biomaterial infiltration by the cells and a faster regeneration process [43]. Adhesion and growth of the osteoblasts on the surface of mat_2_2 was determined by fluorescent staining of cell cytoskeleton and nuclei, followed by CLSM visualization (Figure 5). CLSM images showed that the novel scaffold was favorable to cell growth, since it was covered by numerous well spread (and thus adhered) osteoblasts. On the 4th day of the experiment, the number of cells was higher than on the 2nd day, indicating that mat_2_2 supported cell proliferation. Moreover, osteoblasts cultured on the scaffold had typical flattened morphology and extensive cytoskeleton structure, proving the biocompatibility of the mat_2_2.

All performed cell culture experiments demonstrated that the novel fabrication method allowed for obtaining a scaffold that revealed high osteoconductivity, which is defined as the ability of the biomaterial to support cell adhesion and proliferation [3]. Thus, the developed scaffold appears to have very promising potential in bone tissue engineering applications.

## 4. Conclusions

The present study demonstrated that simultaneous application of relatively simple and cost-effective techniques, such as gas-foaming and freeze-drying, allows for gaining a highly porous structure of biomaterials with predominant open and interconnected porosity. It was demonstrated that the best porosity is obtained when solvent (CH_3_COOH) and foaming agent (NaHCO_3_) are applied at ratio 1:1. Moreover, the highest share of macropores is achieved when the solvent is applied at higher concentrations (1% or 2% *v/v*). Thus, the biomaterial with the most promising biomedical potential and optimal porosity was produced using 2% *v/v* CH_3_COOH and 2% *w/v* NaHCO_3_. The developed scaffold is characterized by high total (70%) and open (50%) porosity, as well as a uniform and interconnected pore network. However, the high porosity of the novel biomaterial decreased its mechanical strength. Importantly, the scaffold is non-toxic and favors osteoblasts adhesion and growth on its surface. Moreover, the biomaterial has apatite-forming ability, which provides good osseointegration. All mentioned properties make the novel biomaterial a promising candidate to be used in regenerative medicine in non-load bearing implantation sites, as well as in use as a scaffold allowing for the generation of bone grafts under in vitro conditions. Nevertheless, further physicochemical and biological studies are required to comprehensively evaluate the biomedical potential of the developed scaffold.

## 5. Patents

The composition and method for the production of the scaffolds was claimed in the Polish patent application no. P.426788.

## Figures and Tables

**Table 1:** Concentrations of solvent (CH_3_COOH) and gas-foaming agent (NaHCO_3_) which were applied during fabrication process of the scaffolds.

Sample Designation	CH_3_COOH (% *v/v*)	NaHCO_3_ (% *w/v*)
Mat_0.5_0.5	0.5	0.5
Mat_0.5_1	0.5	1
Mat_1_1	1	1
Mat_1_1.5	1	1.5
Mat_2_2	2	2
Control	2	none

**Table 2:** Porosity of the biomaterials determined by micro-CT analysis.

Porosity (%)	Control	Mat_0.5_0.5	Mat_0.5_1	Mat_1_1	Mat_1_1.5	Mat_2_2
Total	8.81 ± 1.61	57.46 ± 2.08 *	47.88 ± 7.48 *^#^	59.61 ± 3.00 *	45.79 ± 9.96 *^#^	70.32 ± 2.33 *
Closed	8.56 ± 1.56	33.22 ± 2.65 *	32.48 ± 2.46 *^#$^	22.27 ± 2.20 *	30.16 ± 4.85 *^#$^	19.99 ± 3.01 *
Open	0.24 ± 0.14	24.24 ± 1.16 *	15.40 ± 8.14 ^#$^	37.34 ± 3.78 *	15.63 ± 12.30 ^#$^	50.32 ± 4.28 *

* statistically significant results compared to control sample; ^$^ statistically significant results compared to mat_1_1; ^#^ statistically significant results compared to mat_2_2 (*p* < 0.05, One-way ANOVA followed by Tukey’s test).

**Table 3:** Compressive strength and Young’s modulus values of the produced biomaterials.

Mechanical Parameters (MPa)	Control	Mat_0.5_0.5	Mat_0.5_1	Mat_1_1	Mat_1_1.5	Mat_2_2
Compressive Strength	36.19 ± 8.77	1.32 ± 0.11 *	2.35 ± 0.44 *	0.74 ± 0.03 *	0.77 ± 0.04 *	0.86 ± 0.06 *
Young’s Modulus	179.14 ± 9.38	10.17 ± 1.37 *	11.79 ± 3.45 *	6.04 ± 0.30 *	6.69 ± 0.81 *	7.42 ± 0.16 *

* statistically significant results compared to control sample (*p* < 0.05, One-way ANOVA followed by Tukey’s test).
